# Impact of long-term industrial contamination on the bacterial communities in urban river sediments

**DOI:** 10.1186/s12866-020-01937-x

**Published:** 2020-08-14

**Authors:** Lei Zhang, Demei Tu, Xingchen Li, Wenxuan Lu, Jing Li

**Affiliations:** 1 School of Civil Engineering and Architecture, Chuzhou University, 1 West Huifeng Road, Chuzhou, 239000 China; 2 Fisheries Research Institute, Anhui Academy of Agricultural Sciences, Hefei, 230036 China

**Keywords:** Urban river, Industrial pollution, Bacterial community, Environmental factors, Co–occurrence network

## Abstract

**Background:**

The contamination of the aquatic environment of urban rivers with industrial wastewater has affected the abiotic conditions and biological activities of the trophic levels of the ecosystem, particularly sediments. However, most current research about microorganism in urban aquatic environments has focused on indicator bacteria related to feces and organic pollution. Meanwhile, they ignored the interactions among microorganisms. To deeply understand the impact of industrial contamination on microbial community, we study the bacterial community structure and diversity in river sediments under the influence of different types of industrial pollution by Illumina MiSeq high-throughput sequencing technology and conduct a more detailed analysis of microbial community structure through co-occurrence networks.

**Results:**

The overall community composition and abundance of individual bacterial groups differed between samples. In addition, redundancy analysis indicated that the structure of the bacterial community in river sediments was influenced by a variety of environmental factors. TN, TP, TOC and metals (Cu, Zn and Cd) were the most important driving factors that determined the bacterial community in urban river sediments (*P* < 0.01). According to PICRUSt analysis, the bacterial communities in different locations had similar overall functional profiles. It is worth noting that the 15 functional genes related to xenobiotics biodegradation and metabolism were the most abundant in the same location. The non-random assembly patterns of bacterial composition in different types of industrially polluted sediments were determined by a co-occurrence network. Environmental conditions resulting from different industrial pollutants may play an important role in determining their co-occurrence patterns of these bacterial taxa. Among them, the bacterial taxa involved in carbon and nitrogen cycles in module I were relatively abundant, and the bacterial taxa in module II were involved in the repair of metal pollution.

**Conclusions:**

Our data indicate that long-term potential interactions between different types of industrial pollution and taxa collectively affect the structure of the bacterial community in urban river sediments.

## Background

Urban rivers are a vitally important foundation for reclaimed water utilization and the development of urban environments [1]. However, rivers flowing through cities often receive treated and untreated urban wastewater [2]. Among them, industrial wastewater has become one of the important sources of urban river aquatic environment pollution [3]. Exceeding a certain content of organic and inorganic contaminants in industrial wastewater will not only have negative consequences for aquatic ecosystems [4–6] but also pose significant health risks for water users [2]. The discharge of improperly treated wastewater into rivers or reservoirs used for agricultural irrigation may also have indirect adverse impacts on human health [7]. In addition, refractory organics and heavy metals released from industrial wastewater can cause long-term problems.

As an important part of the river system, sediments are complex habitats densely settled by various microorganisms [3]. Microorganisms are considered to be the most diverse and abundant biogroup in the world, regulating global biogeochemical cycles and affecting the functions of almost all ecosystems [8–10]. When different types of industrial wastewater are continuously discharged into the water environment, contaminants in the water can be diluted or purified. Conversely, contaminants in sediments can exist for longer periods of time [11]. Meanwhile, microbial communities in freshwater sediments are highly sensitive to changes in physicochemical status [12]. This inevitably affects the microbial community and its function in the sediments [13]. To adapt to different survival environments, microorganisms usually form a specific community structure to address various adverse effects [14]. Hence, analysis of the microbial communities in polluted river sediments can better identify potential biological indicator species as well as biomarker communities that respond to specific pollutants [15].

Many previous studies have analyzed the diversity and composition of microbial communities in rivers and determined the microbial indicators under local environmental impacts. For example, different bacterial indicators in river sediments under different environmental conditions were determined by linear discriminant analysis effect size (LEfSe) analysis [16, 17]. In addition, indicator species analysis methods were used to determine bacterial indicators in rivers under different land use types and human activity gradients [18]. However, most of these studies focused only on the interaction between river microorganisms and environmental pressure and ignored the interactions among microorganisms. In fact, microbe-microbe interactions have a crucial impact on community assembly and ecosystem function [19, 20]. In addition, the dynamics and composition of microbial communities are also greatly influenced by abiotic environmental factors [21, 22]. Therefore, understanding the interactions between microorganisms can provide new insights for further studying the structure of microbial communities in sediments under different environmental conditions.

Microbial co-occurrence networks can not only analyze microbe-microbe interactions and keystone species in the ecosystem but also explain potential intra or interspecific interactions in the water environment [23]. At present, co-occurrence networks have been applied to study complex ecosystems, such as marine bacterioplankton [24] and soil bacterial [25] or fungal communities [26]. Co-occurrence networks have intrinsic power and are useful in revealing information about community organizations, interactions among members, keystone species and their responses to different environmental conditions [27]. At the same time, the application of functional prediction technology can provide a glimpse of the overview of the functional spectrum of the microbiome, making the study of bacterial communities more detailed [28].

In fact, despite our advances in freshwater microbial ecology, most microbial research in urban aquatic environments has focused on indicator bacteria associated with feces and organic pollution [29–31]. Research on the effects of different types of industrial pollution on microorganisms in the aquatic environment of urban rivers is even more limited. Here, we study the bacterial community structure and diversity in river sediments under the influence of different types of industrial pollution by Illumina MiSeq high-throughput sequencing technology and conduct a more detailed analysis of microbial community structure through co-occurrence networks. We hypothesized that the sediment bacterial community is influenced by the river environment, including nutritional factors and heavy metals. Industrial pollution influences the bacterial community structure in river sediment by changing the river environment. Our specific goals are (1) to explore the causes for the differences in the composition of bacterial communities in river sediments affected by different types of industrial pollution; (2) to explore the relationship between bacterial community function and habitat in river sediments under different industrial pollution conditions.

## Results

### Physicochemical characteristics of sediment samples

**Fig. 1:**
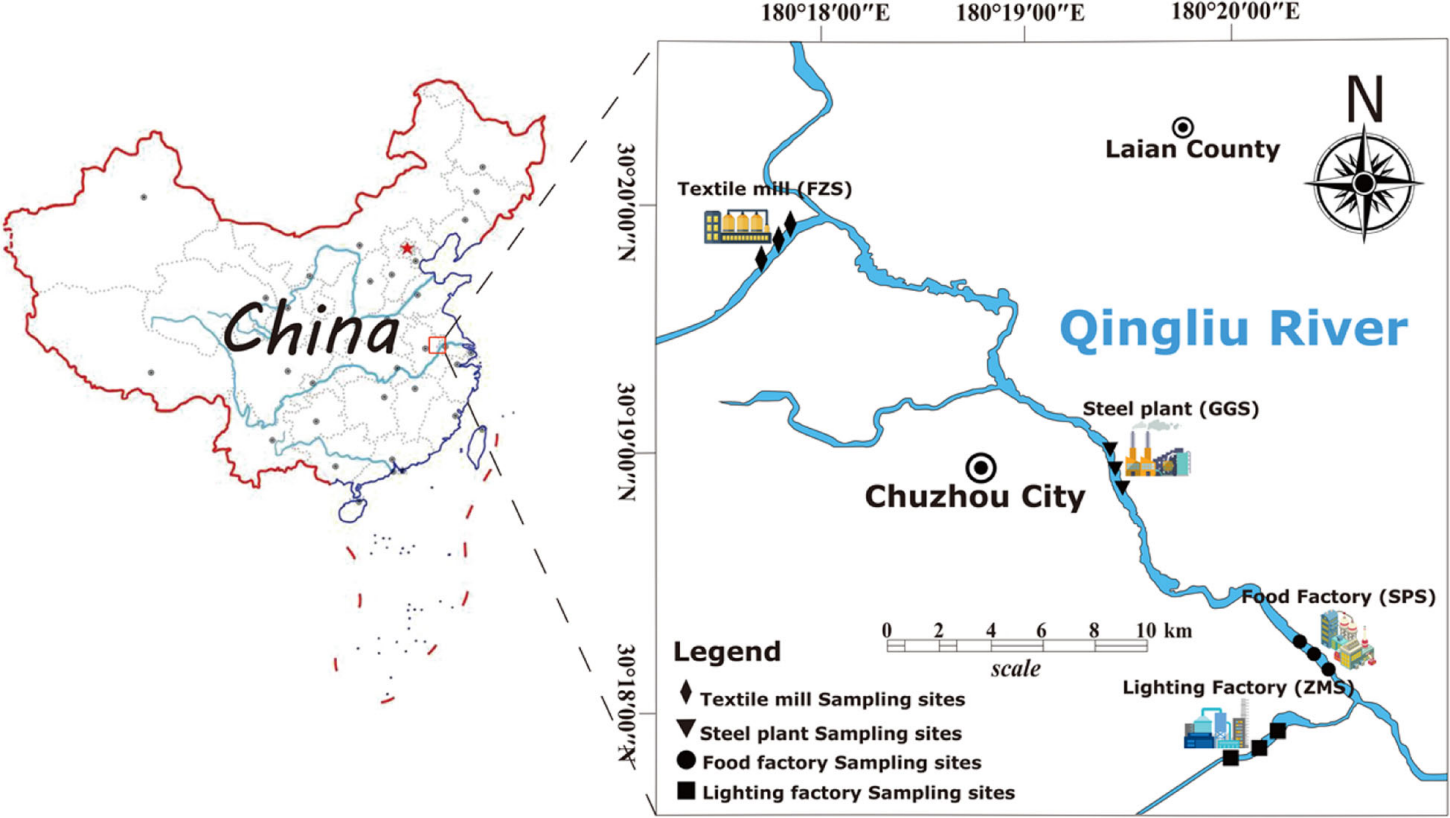
Location of sampling sites in Qingliu River, Anhui, China

Table S1 shows the 12 physicochemical properties of different types of industrial contaminated surface water and sediments from the four sample sites (Fig. 1). The temperature and pH values of all samples were between 21.1 ~ 22.7 °C and 7.19 ~ 10.01, respectively. The TN (total nitrogen), TP (total phosphorus), and TOC (total organic carbon) contents of GGS (steel plant) were higher than those of other samples (Table S1). Additionally, the heavy metal concentrations in the four sites were compared. The results showed that Cu and Zn levels in GGS were the highest (Table S1). The highest contents of Pb and Cr were observed in ZMS (lighting factory), and the highest content of Cd was observed in FZS (Table S1). Based on the Chinese Soil Environmental Quality Standard (GB15618–1995), the concentration of heavy metals is divided into five classes (I, II, III, IV, V), corresponding to clean, relatively clean, normal, polluted, and moderately to heavily polluted, respectively [32]. The Cd concentration reached the class IV standard in ZMS and SPS and the class V standard in FZS (Table S2).

### Diversity and richness analysis of bacterial communities

**Fig. 2:**
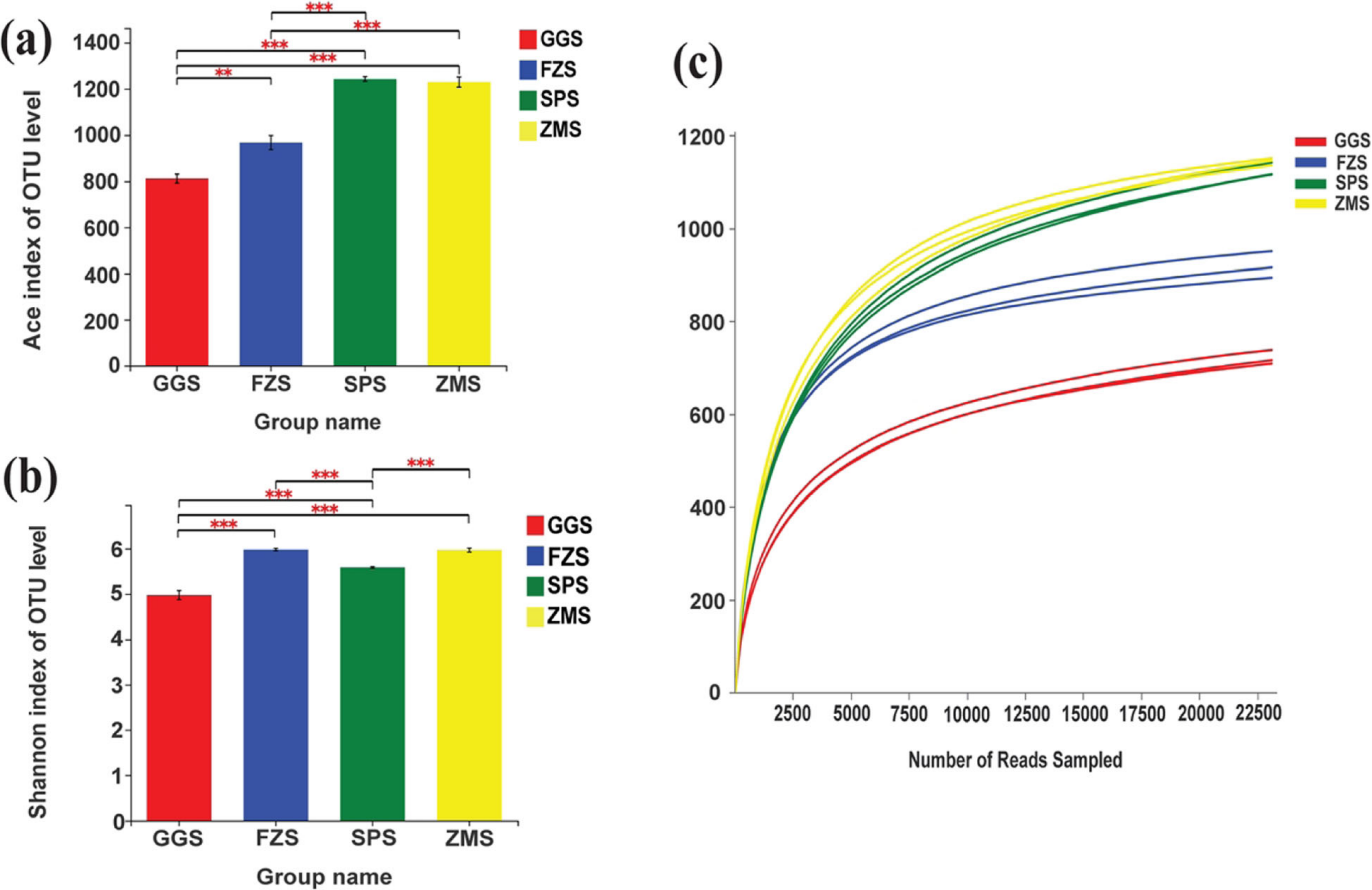
Alpha diversity of sediment samples from different types of industrial pollutions. **(a)** Richness, indicated by Ace, Ace species richness estimator: higher values represent higher diversity. **(b)** Diversity, indicated by Shannon, the Shannon index: higher values represent higher diversity. * *P* < 0.05, ** *P* < 0.01, *** *P* < 0.001. **(c)** Rarefaction curves of OTUs clustered at 97% sequence identity across the twelve samples

In this work, we analyzed microbial community diversity and phylogenetic structure in samples from different types of industrially polluted sediments by Illumina MiSeq high-throughput sequencing. After trimming, screening, and removing chimeras and single pieces, 505,911 valid 16S rRNA sequences were obtained. Among them, at least 31,412 valid sequences were obtained for each sample, with an average length of 417 bp. OTUs (operational taxonomic units) were grouped at the 97% cutoff, and diversity indexes and richness estimates were calculated for each sediment sample (Table S3). The Chao and ACE indexes of different sediment samples were compared, and the results were consistent with the above results (Fig. 2 (a); Table S3).

Simpson and Shannon index analysis results showed that FZS and ZMS had the highest diversity, followed by SPS, whereas GGS displayed significantly lower diversity (Table S3; Fig. 2 (b)). All rarefaction curves approached saturation, indicating sufficient sequencing depth (Fig. 2 (c)). Similarly, the coverage of each sample was higher than 0.99 (Table S3), which indicates that the sequencing depth was sufficient and some rare species were included.

### Composition of bacterial communities

**Fig. 3:**
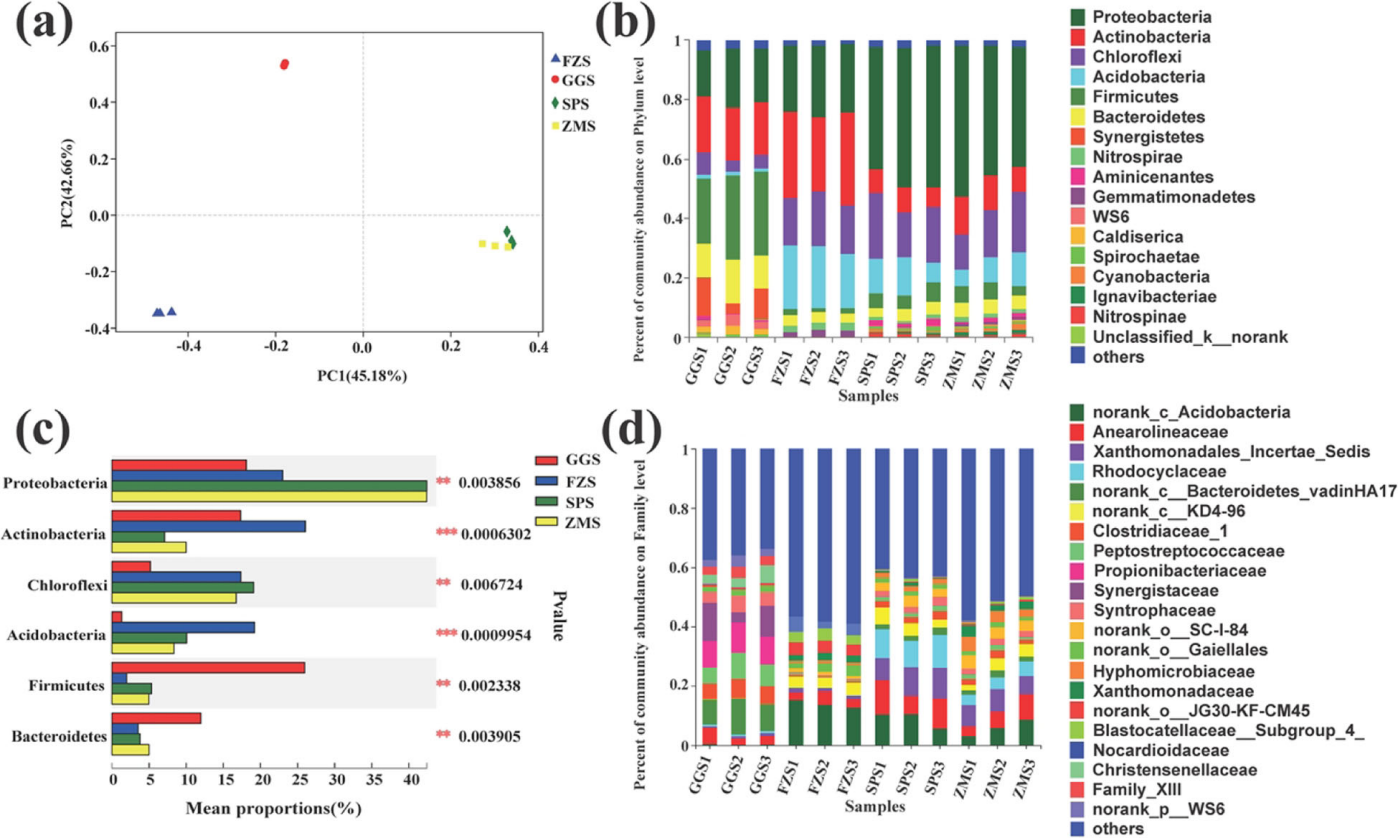
Principled coordinate analysis (PCoA) for the four groups of different industrial polluted sediment communities at the OTU level (**a**). Community composition of bacteria in the phylum level (**b**) and the family level (**d**) in sediment samples. (**c)** Significantly different relative abundance of dominant bacteria in four groups of different industrial polluted sediments. One-way analysis of variance (ANOVA) was used to evaluate the importance of comparisons between indicated groups. * *P* < 0.05, ** *P* < 0.01, *** *P* < 0.001

The overall characteristics of bacterial communities in different types of industrially polluted sediment samples were observed by principal coordinate analysis (PCoA) (Fig. 3 (a)). PC1 and PC2 accounted for 45.18 and 42.88%, respectively. In general, spatially adjacent samples were more closely distributed. Therefore, we found that SPS and ZMS samples had similar features. ANOSIM analysis (analysis of similarities) confirmed significant differences in bacterial community structure in different types of industrially polluted sediment samples (R = 1, *p* = 0.001) (Table S4).

A total of 16 major bacterial phyla were detected in sediment samples collected at different locations. Among them, *Proteobacteria*, *Actinobacteria*, *Chloroflexi*, *Acidobacteria*, *Firmicutes* and *Bacteroidetes* together accounted for 77.62 to 94.49% of OTUs in all samples, and their relative abundance in each sample was higher than 1% (Fig. 3 (b)). These dominant bacterial phyla exhibited significant differences in different types of industrially polluted sediment (Fig. 3 (c)). Further analysis revealed that there was no significant difference in the relative abundance of the dominant bacterial phyla in both SPS and ZMS sediments (Fig. S1). *Proteobacteria* had the highest average relative abundance in sediment samples from SPS and ZMS, accounting for 45.22 and 45.04%, respectively. The average relative abundance of *Firmicutes* was highest in GGS (26.59%), while the phylum with the highest average relative abundance in FZS was *Actinobacteria* (28.41%).

To better explain the structure of the microbial community in different types of industrial polluted sediments, the relative abundance and classification of OTUs were analyzed at the family level (Fig. 3 (d)). It is worth noting that some species had a large proportion in some samples and were the dominant bacteria, while in other samples, its proportion was small, or even nonexistent. Family *Propionibacteriaceae* (9.51%) and family *Synergistaceae* (8.96%) were the dominant family in GGS samples. However, they were almost undetectable in the other three groups of samples. In FZS samples to *norank_c_Acidobacteria* (13.81%) are the most common, other dominant families including *norank_o_JG30 KF-CM45* (4.04%), *norank_CKD4–96* (3.81%) and family *Nocardioidaceae* (3.77%). Among them, the family *Nocardioidaceae* was found to be much more abundant in the FZS samples than in the other samples. The most abundant families in SPS and ZMS were family *Rhodocyclaceae* (9.99%) and family *Xanthomonadales_Incertae_Sedis* (6.99%).

### Significant differences in microbial communities

**Fig. 4:**
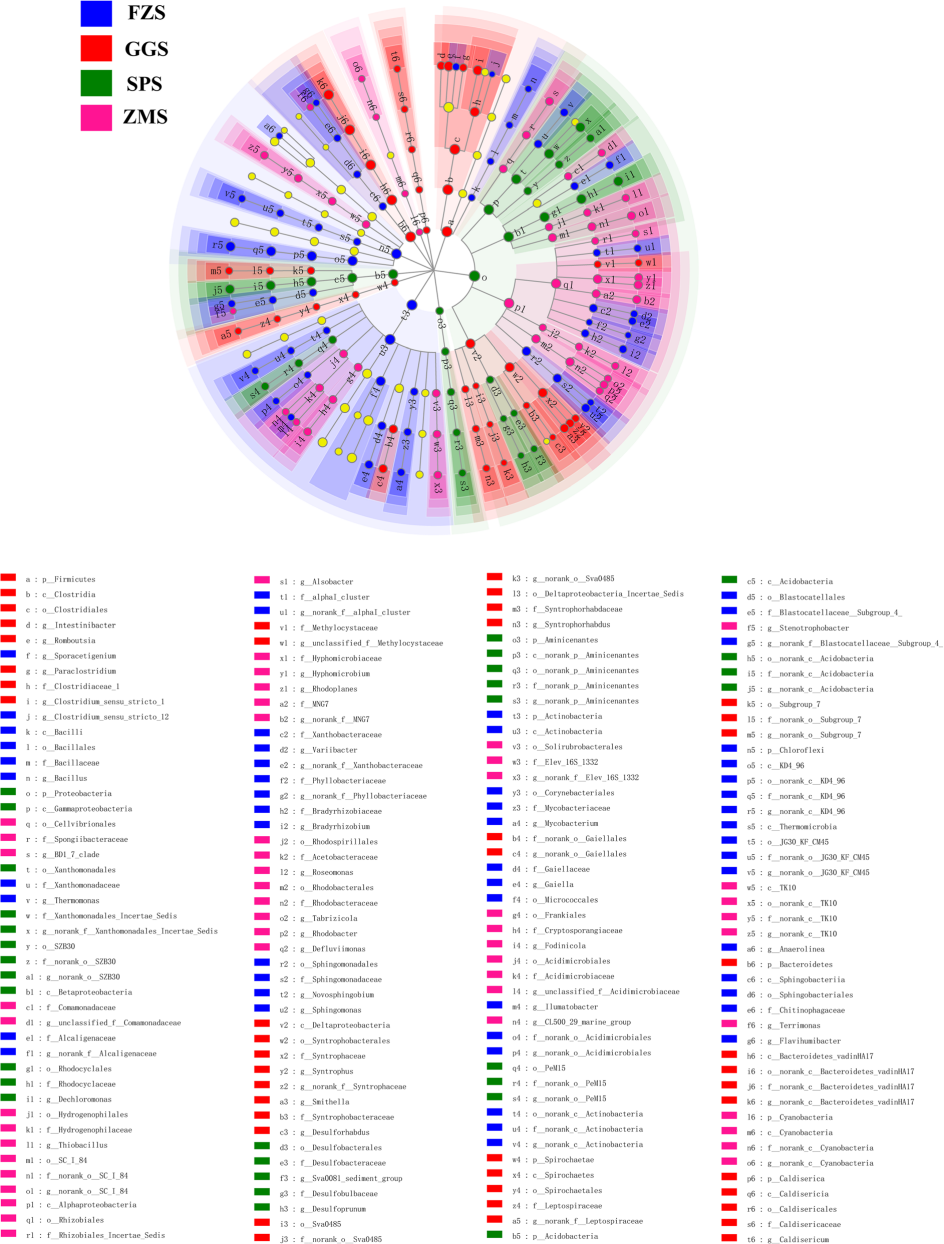
Cladogram showing the phylogenetic distribution of the bacterial lineages in river sediments under different industrial pollution. The phylum, class, order, family, and genus levels are listed in order from the inside to the outside of the cladogram, used to determine the most likely to explain the difference between taxa groups. Different-colored nodes correspond to different sample groups, which represent taxa with significant enrichment in the corresponding group and significant influence on intergroup difference. Yellow circles stand for taxa with no significant differences in the sediment

Biomarker analysis with linear discriminant analysis (LDA) effect size (LEfSe) was used to identify species with significant abundance differences in different types of industrially polluted sediment samples. As showed in Fig. 4, 176 bacterial clades present statistically significant differences with a LDA threshold of 3.5 (Fig. S2). The degree of enrichment was greatest in FZS for most bacteria, and a total of 54 clades showed an abundance advantage. In contrast, the degree of enrichment was smallest in the SPS samples, with only 30 clades. It is worth noting that the family *Rhodocyclaceae* (LDA = 4.92) and genus *Dechloromonas* (LDA = 4.91) in SPS not only have high relative abundance but also have significant effects on differences among groups. In contrast, although the relative abundance of the family *Sphingomonadaceae* (LDA = 4.51) and genus *Sphingomonas* (LDA = 4.51) in FZS was low, they also had significant effects on differences among groups.

### Environmental factor analysis

**Fig. 5:**
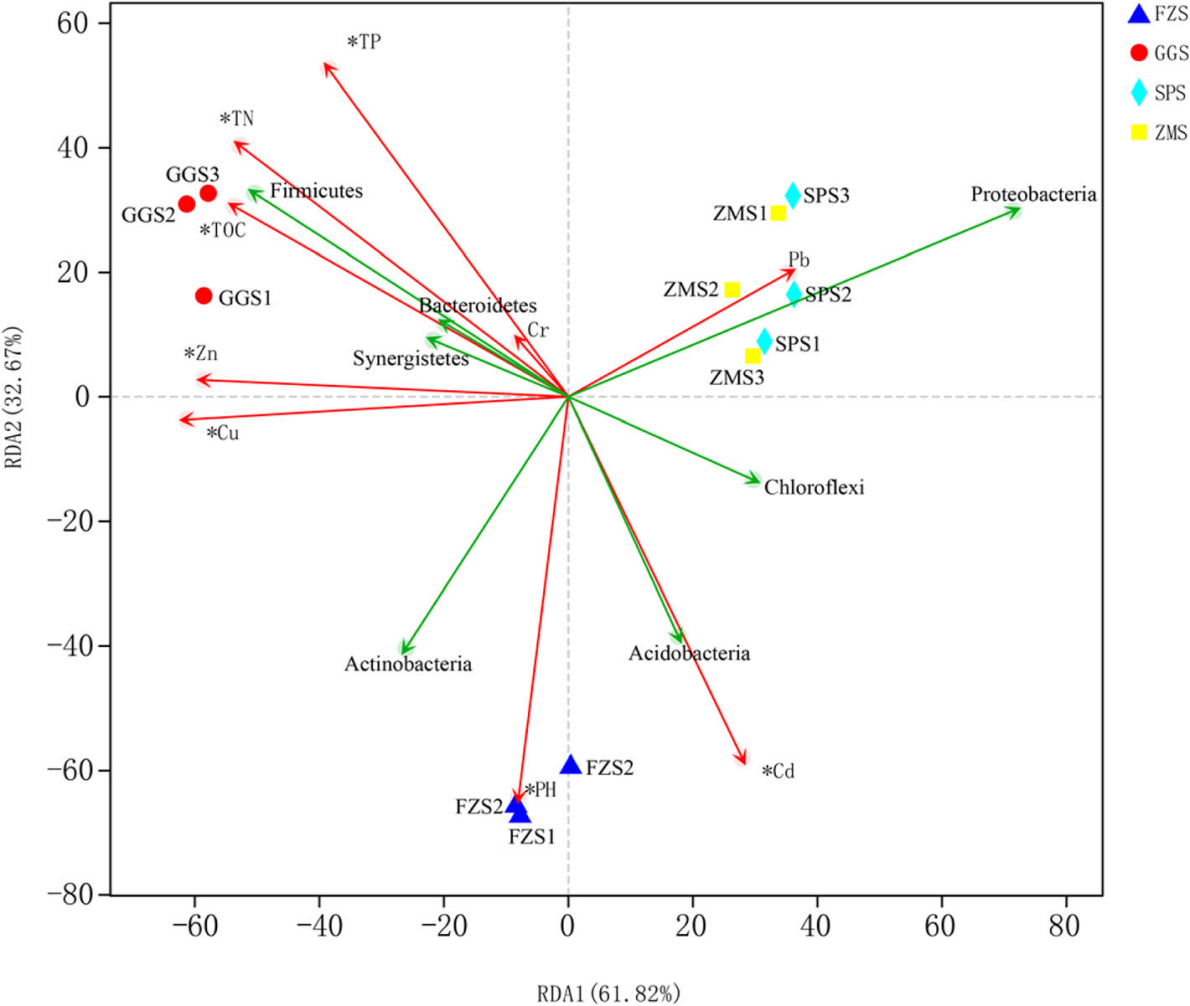
Redundancy analysis (RDA) of bacterial community composition in sediments and environmental factors from different types of industrial polluted sediments. (note that the significant environmental factors identified by the Monte-Carlo test are marked with an asterisk. An * represents significant correlations at *P* < 0.01)

The correlation between environmental parameters and bacterial community composition was determined by RDA (redundancy analysis) (Fig. 5). TN, TP, TOC, pH, Cu, Zn and Cd were environmental variables that had a significant effect on the relationship between the bacterial community and the environment (all *P* < 0.01). Among them, Cu and Zn were positively correlated with samples from GGS, and Cd was also positively correlated with the FZS samples. Moreover, *Firmicutes* was positively correlated with TN, TP, TOC, Cu and Zn. pH and Cd were also positively correlated with *Actinobacteria* and *Acidobacteria*. However, *Proteobacteria* and *Chloroflexi* were significantly negatively correlated with TN, TP, and TOC.

### Co-occurrence network analysis

**Fig. 6:**
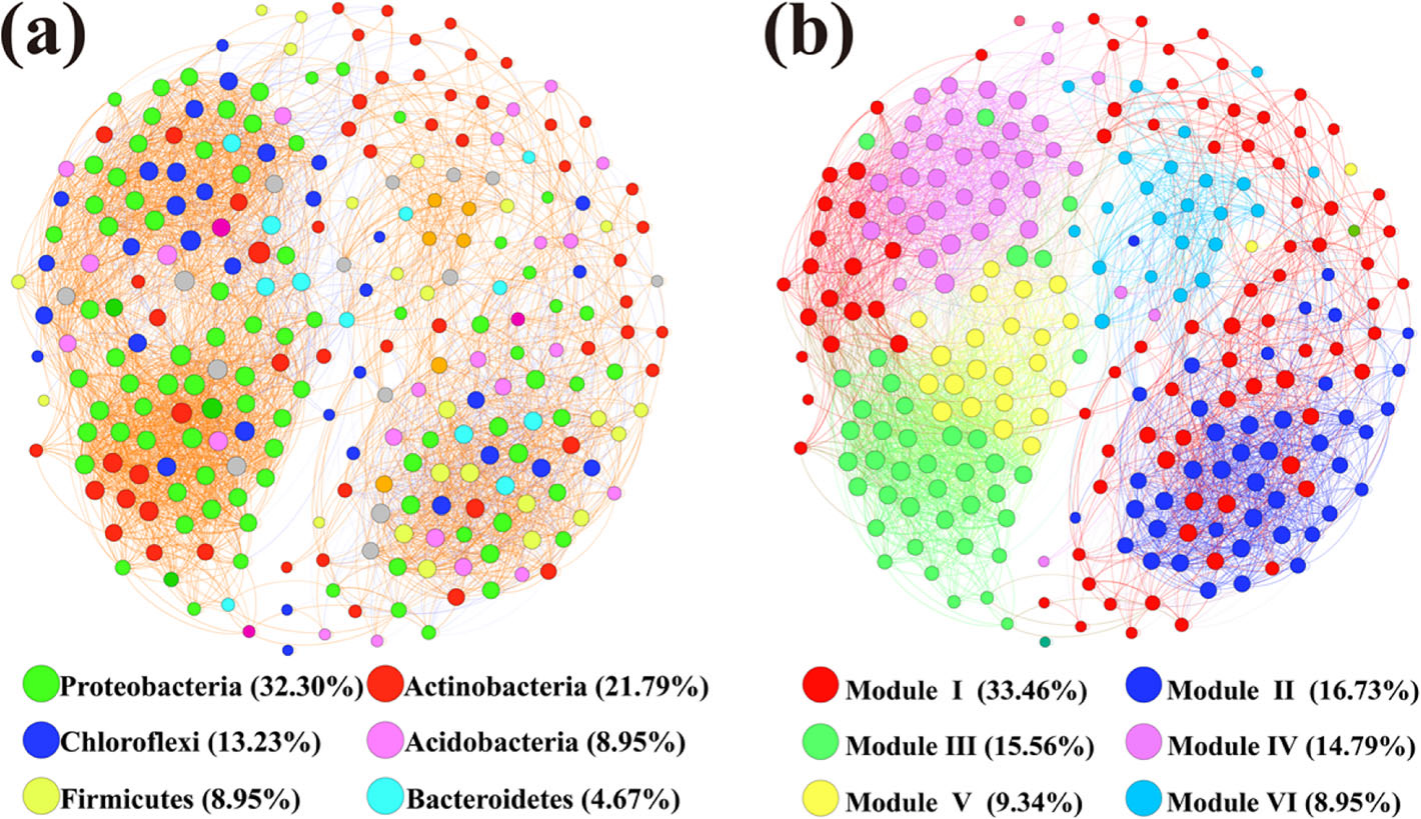
Co-occurrence networks of bacterial communities in different types of industrial polluted sediments based on correlation analysis. The nodes in network (**a**) are colored by phylum. The nodes in network (**b**) are colored by modularity class. The connections indicate strong (spearman’s ρ ≥ 0.7) and significant (*P* < 0.05) correlations. The size of each node is proportional to the relative abundance of specific genus

Considering the non-random aggregation pattern of microbial communities in river sediments under different types of industrial pollution, a network interface was established to better show the topological and taxonomic characteristics of microbial co-occurrence patterns (Fig. 6). According to the analysis results, 4196 edges were captured among 257 nodes that described significant correlations between species (ρ > 0.7, *P* < 0.05). At the same time, significant topological characteristics were derived based on calculations to determine the complex patterns of interrelationships among nodes [33]. The average path length (APL) was 2.825 edges, and the diameter was 6 edges. The clustering coefficient (CC) was 0.641, and the modularity index (MD) was 0.827, where MD > 0.4 indicates that there was a modular structure in the network [34].

The nodes in the network were divided into 6 bacterial phyla (Fig. 6 (a)). Among them, *Proteobacteria*, *Actinobacteria*, and *Chloroflexi* accounted for 63.2% of all nodes, and they were also dominant bacterial phyla in the community. When the node distribution was modularized, all nodes were mainly divided into six modules (Fig. 6 (b)). Each module consisted of a set of OTU nodes, and the interconnections among these nodes were more frequent than the nodes in other modules. The OTUs in module I had a higher relative abundance in FZS, and the OTUs in module II and module VI had a higher relative abundance in GGS. Module III and module V had a higher relative abundance in ZMS, and module IV dominated in SPS. The top five keystone genera with the highest number of connections were *Gaiella*, *Denitratisoma*, *Anaeromyxobacter*, *Candidatus_Microthrix*, and *unclassified_p__Chloroflexi*, and the number of related genera was not less than sixty. In addition, the betweenness centrality values of the genera *Denitratisoma*, *Anaeromyxobacter*, and *Candidatus_Microthrix* were all less than 200 (their closeness centrality range from 0.379 to 0.382). This represents a high degree and low betweenness centrality values, indicating that they can be considered central species [20, 35].

### PICRUSt functional predictive analysis

**Fig. 7:**
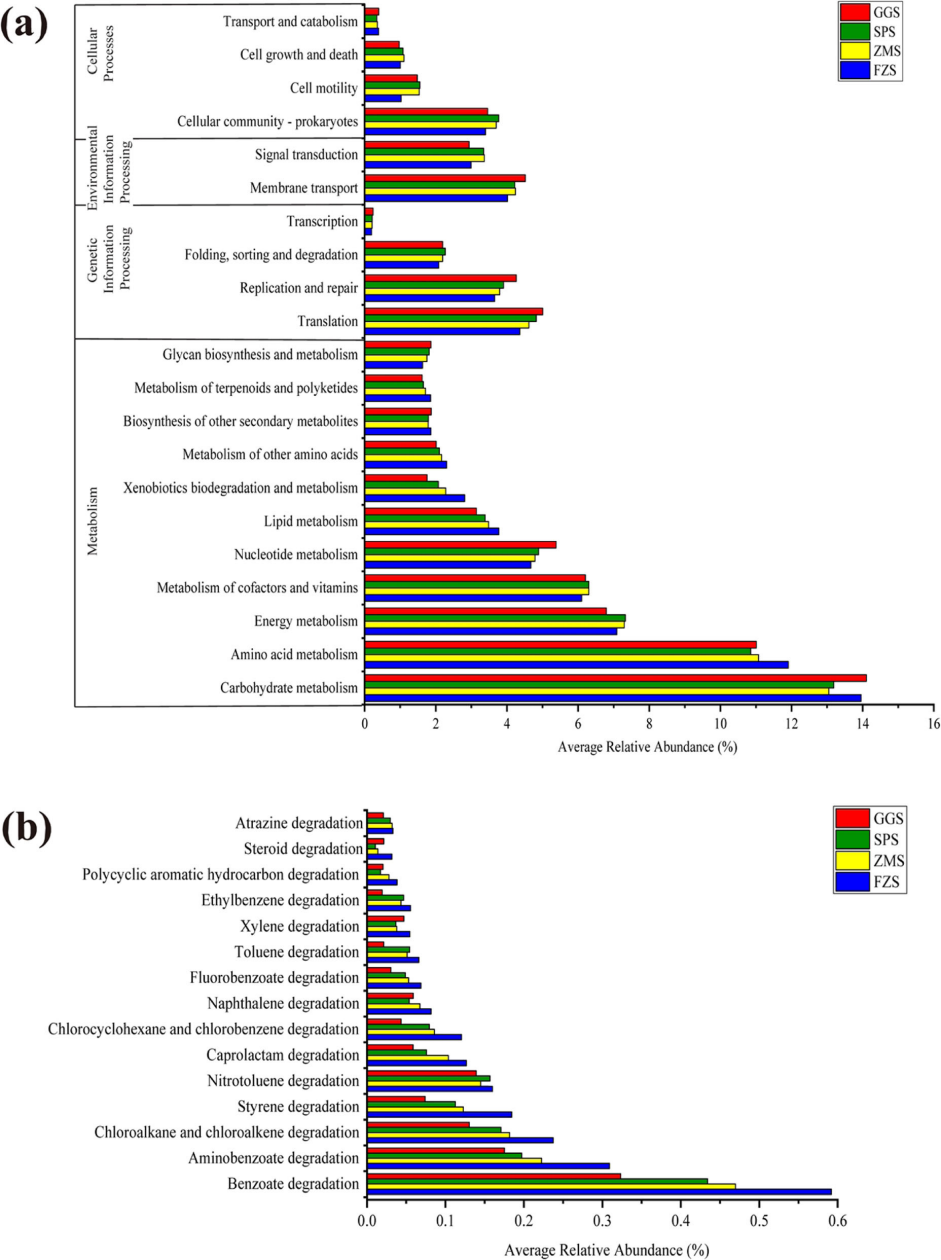
The comparison of bacterial community functions predicted by PICRUSt at level 2 and level 3 among different groups. **(a)** The predictive functions at level 2 is related to metabolism, genetic information processing, environmental information processing and cellular processes. **(b)** The predictive functions at level 3 is related to xenobiotics degradation and metabolism

PICRUSt (phylogenetic investigation of communities by reconstruction of unobserved states) analysis was conducted to predict the metabolic functions of bacterial communities. The results indicated that the major functional gene families were related to metabolism, genetic information processing, environmental information processing and cellular processes (Fig. 7(a)). Among them, the relative abundance of metabolism was the highest in all samples, followed by genetic information processing. Their average proportions in different groups were 68.94 and 10.96%, respectively. At KEGG (Kyoto Encyclopedia of Genes and Genomes) level 2, a large number of sequences in each sediment sample were allocated to carbohydrate metabolism and amino acid metabolism (Fig. 7 (a)). In addition, rivers are the primary recipient of pollutants and xenobiotics input from the watershed, most aquatic organisms, as well as bacteria, are exposed to these xenobiotics [36]. Therefore, we focused on the functional genes of xenobiotics biodegradation and metabolism. In this category, FZS had the highest average relative abundance (Fig. 7(a)). At KEGG level 3, 15 individual KEGG pathways from xenobiotics biodegradation and metabolism were chosen (Fig. 7 (b)), and the content of each pathway in FZS was higher than the other three groups. Among them, the average relative abundance of benzoate degradation was the highest, followed by aminobenzoate degradation.

## Discussion

Bacteria are important for ensuring and maintaining the environmental and ecological processes of river ecosystems [37]. Here, the bacterial communities in river sediments under different types of industrial pollution were analyzed by Illumina high-throughput sequencing technology. Overall, the sediment bacterial communities were dominated by *Proteobacteria*, *Actinobacteria*, *Chloroflexi*, *Acidobacteria*, *Firmicutes* and *Bacteroidetes* (Fig. 3 (b)). Wang et al. (2016) studied the community structure of aquatic bacteria in urban rivers and obtained similar results [38]. Su et al. (2018) investigated the bacterial communities in coastal sediments and found that *Proteobacteria*, *Firmicutes*, *Chloroflexi*, *Acidobacteria*, *Bacteroidetes*, *Actinobacteria*, *Nitrospirae*, *Gemmatimonadetes* and *Planctomycetes* predominated [39]. These results showed that these sediment samples shared the characteristic profile of high bacterial rank commonly found in other aquatic ecosystems.

Although there were similarly high taxonomic level characteristics between different sampling sites, the relative abundance was different among different sites (Fig. 3 (c)). According to the data from this study, *Proteobacteria* were the most abundant in SPS (food factory) and ZMS (lighting factory) and were mainly composed of *Betaproteobacteria* and *Gammaproteobacteria* (Fig. 4). Most *Proteobacteria* groups play very important roles in the decomposition of organic matter and circulation [40]. Further research found that the family *Rhodocyclaceae* (class *Betaproteobacteria*) not only dominates in SPS but also had significant differences in abundance at different sites (Fig. 4). This strain has extensive metabolic capabilities and can degrade multiple carbon sources, such as many aromatic compounds [41]. Therefore, some members of the family are active in the degradation of recalcitrant chemicals [42]. For example, the genus *Dechloromonas* is capable of degrading a variety of complex organic pollutants (Fig. 4) [43]. Therefore, the dominance of the family *Rhodocyclaceae* in SPS may indicate that the area was rich in organic wastewater compounds.

The most dominant phylum in FZS (textile mill) was *Actinobacteria*. *Actinobacteria* have been confirmed to play a pivotal role in the carbon cycle of freshwater ecosystems [44]. In fact, the data from this study showed that TOC was the lowest in FZS, and nutrition in this sample was relatively poor (Table S1). The results were consistent with previous studies, suggesting that *Actinobacteria* are indeed active in oligotrophic environments [45]. In addition, the family *Sphingomonadaceae* was significantly enriched in FZS, and there were significant differences among different sites (Fig. 4). The family *Sphingomonadaceae* is usually found in habitats contaminated by a high proportion of recalcitrant (poly) aromatic compounds of natural or anthropogenic origin [46–48]. Moreover, the genus *Sphingomonas* in *Sphingomonadaceae* can degrade various recalcitrant compounds (Fig. 4) [49]. Thus, the members of this genus can grow vigorously in polluted environments [50]. It is worth noting that the textile industry produces a large amount of wastewater, which contains a variety of chemical compounds, such as azo dyes, heavy metals, and surfactants [51]. Therefore, the relative advantages of the family *Sphingomonadaceae* in FZS indicated that they are well adapted to the environment in FZS and possibly utilize a wide variety of nutrients to resist or withstand environmental disturbances.

*Firmicutes* are well known for having many members that are able to degrade very recalcitrant organic compounds [43]. Few previous studies also found a dominance of *Firmicutes* in freshwater sediments [52]. However, the abundance of *Firmicutes* was highest in GGS (steel plant). The data from this study indicated that the content of TN, TP and TOC in GGS was the highest, especially the TOC, which was 2 to 3 times that of the other three groups of samples (Table. S1). Meanwhile, RDA showed that the abundance of *Firmicutes* was positively correlated with TN, TP, and TOC (Fig. 5). This is the same result as previous studies, suggesting that as copiotrophs or fast-growing organisms, *Firmicutes* can exist in carbon-rich environments that meet their high energy requirements and maintain their growth rates [53]. Moreover, *Clostridiales* are known as metal-coping bacteria and thrive in environments rich in metal contaminants such as GGS [54] (Fig. 4; Table S1). Therefore, the enrichment of *Clostridiales* may also be one of the reasons for the largest proportion of *Firmicutes* in GGS. These results indicated that the environmental conditions may select different bacterial species, which leads to different spatial distributions of bacterial populations.

Multiple studies have shown that environmental factors, such as temperature [38], nutrients [55], pH [56], water turbidity [57] and sediment particle size [58], often affect the composition and structure of bacterial communities. On the one hand, RDA showed that TN, TP and TOC were significantly related to the composition of bacterial communities in different types of industrial polluted sediments (*P* < 0.01) (Fig. 5). TN, TP, and TOC are important factors for structuring bacterial communities in river sediments, which is consistent with other research results. In reality, microorganisms may prefer to use bioavailable forms of phosphorus, nitrogen, and carbon (e.g., PO_4_^3−^, NO_2_^−^, NO_3_^−^, NH_4_^+^, etc.) and the forms may be more closely related to bacterial community composition [45, 59, 60]. Therefore, the relationship between microbial communities and detailed environmental factors needs to be studied in greater depth. On the other hand, the heavy metals Cu, Zn, and Cd were significantly correlated with the distribution of bacterial communities in different types of industrially polluted sediments (*P* < 0.01) (Fig. 5). It has been documented that high concentrations of heavy metals can significantly reduce bacterial biomass in sediments [61]. This was consistent with our observations that the species richness and diversity in the GGS samples, which had the highest heavy metal concentrations, were the lowest (Fig. 2 (a, b); Table S1).

In summary, the differences in bacterial community composition in different types of industrially polluted sediments reflect the tolerance of OTUs to specific environments. The above abiotic factors, such as TN and TP, may directly change the composition of bacterial communities by affecting the growth of certain bacteria in the sediment. That is, changes in the physicochemical properties of river sediments caused by the input of different types of industrial wastewater drive the formation of different bacterial communities. Meanwhile, this also means that microbial communities in urban river sediments have potentially evolved phylogenetic versatilities under the long-term effects of industrial pollution.

The interrelationships among different microbial communities play a pivotal role in maintaining the structure, function and stability of microbial ecosystems [14]. In the network analysis of this study, most nodes belonged to three dominant phyla: *Proteobacteria*, *Actinobacteria* and *Chloroflexi* (Fig. 6 (a)). The genera *Gaiella*, *Denitratisoma*, *Anaeromyxobacter*, *Candidatus_Microthrix*, and *unclassified_p__Chloroflexi* were the top five with the highest number of connections. This suggested that the other genera respond more strongly to the metabolites produced by these five genera [62]. At the same time, because these bacterial taxa have highly connected nodes, they are recognized as keystone taxa here [14, 63]. Compared with other taxa in the network, keystone taxa play an important role in maintaining the network structure [22]. It is speculated that the disappearance of keystone taxa may lead to disintegration of the network [44]. Keystone taxa are by the definition the taxa essential for ensuring and maintaining stability, so their existence is by definition important for the stability of ecosystem structure and function. Additionally, the average relative abundances of the genera *Gaiella*, *Denitratisoma*, *Anaeromyxobacter*, *Candidatus_Microthrix*, and *unclassified_p__Chloroflexi* were all low (0.24% ~ 0.65%), suggesting the significance of rare genera in bacterial communities. Currently, rare genera are being increasingly recognized as crucial components of communities in biochemical processes and community assemblies [63]. Although the abundance of such genera may not have been high, more attention should be paid to them as key nodes in the microbial community [14].

In addition, the genera *Denitratisoma*, *Anaeromyxobacter*, and *Candidatus_Microthrix* were considered central species due to their high degree (> 60) and low betweenness centrality values (< 200). The genus *Denitratisoma* contains denitrifying bacteria that contribute to the removal of nitrogen [64]. The genus *Anaeromyxobacter* is metal-reducing bacteria, and members of the bacteria can affect the mobility of metal contaminants [65, 66]. Moreover, previous studies have shown that the genus *Candidatus_Microthrix* helps in the removal of total nitrogen [67]. Therefore, these keystone taxa may play a pivotal role in ecological function processes.

Due to the modularity, the entire network was mainly divided into six modules (Fig. 6 (b)). Modularity may reflect habitat heterogeneity and divergent selection regimes [68]. Meanwhile, the habitat preference of microorganisms may also help determine their co-occurrence patterns [19]. Therefore, it can be reasonably found that microorganisms in different types of industrial polluted sediments tend to form distinct modules. The main taxa in module I may be bacteria involved in the biogeochemical C- and N-cycles. For instance, the genus *Nocardioides* can utilize multiple organic compounds as carbon source [69]. The genus *Nitrospira* consists of chemically autotrophic nitrite-oxidizing bacteria [60]. The genus *Streptomyces* has been shown to participate in the nitrogen cycle [70]. Other bacteria in module I included *Microbacteriaceae*, *Rhodospirillaceae*, *Bradyrhizobiaceae* and *Streptomycetaceae*, which are also involved in C and N cycling [71]. Apparently, these bacteria had the highest abundance in FZS, indicating that carbon and nitrogen cycling associated with microorganisms occurs frequently in this area. Sulfate-reducing bacteria (SRB) in module II, including the genera *Clostridium_sensu_stricto_1*, *Defluviicoccus*, and *Desulfobulbus*, were significantly enriched in GGS [72]. SRB is a group of anaerobic microorganisms that can reduce sulfate to hydrogen sulfide, and hydrogen sulfide can quickly react with heavy metals to form a stable precipitate [73, 74]. Therefore, SRB plays a major role in repairing the environment polluted by heavy metals (Fe, Cu, Pb, Zn, etc.) [72]. Therefore, heavy metals in GGS may be one of the important factors driving these bacteria. In conclusion, the non-random assembly pattern of these bacteria indicates that the complexity of bacterial community structure and functional processes in river sediments under different types of industrial pollution seems to be dominated by environmental filtering and function-driven.

According to PICRUSt analysis, the overall functional profiles of bacterial communities in different river sediment samples were similar. Carbohydrate metabolism and amino acid metabolism were the dominant metabolic genes in the bacterial community (Fig. 7(a)). As core resources metabolism pathways, they were potential drivers of microbial community structure and function of microbial communities in rivers [36]. It is worth noting that the xenobiotics biodegradation and metabolism that we focus on had more advantage in FZS (Fig. 7(a)). The possible reasons were FZS accepted more xenobiotic compounds from industrial wastewater, and these compounds were used by bacteria as the sources of carbon, nitrogen, or energy. Some previous reports indicated that there was a correlation between the abundance of xenobiotic degradation genes and the rate of xenobiotic biodegradation [75, 76]. Therefore, biodegradation genes could be proposed as indicators of the presence of xenobiotics and their metabolites [45, 77]. Furthermore, FZS surpassed the other three groups in the functional profiles related to the degradation of 15 chemical pollutants (Fig. 7(b)). For instance, benzoate degradation and aminobenzoate degradation. Meanwhile, the enrichment of many organic pollutant-degrading bacteria was found in FZS, such as genera *Sphingomonas*, *Mycobacterium*, *Novosphingobium*, and *Bacillus* (Fig. 4) [78–80]. The predicted higher enrichment of chemical pollutant degradation pathways means that the pollution was more severe in FZS, while such functional response of bacterial community might accelerate the bioremediation of contaminated zone. In general, the PICRUSt algorithm determined the predicted functions of the microbial community, providing a general overview of the functional potential within the community. However, rarefaction of pooled DNA samples fails to capture the full extent of diversity present within the system, which is likely reflected in the predicted functional profile [81]. Furthermore, this method is influenced by phylogenetic differences between environmental samples and sequenced genomes [28]. Therefore, we propose that further studies are needed in this system that use metagenomic sequencing and marker gene studies, to fully assess gene categories.

In this study, because the pollutants at each sampling point have been discharged into the water through pipes or channels for a long time, the sampling conditions were restricted. Therefore, we only paid attention to the impact of long-term discharge of industrial wastewater on the bacterial community of river sediments, and we did not cover the research before wastewater discharge. This may cause some limitations to our research. However, our findings represent an important step in understanding the impact of long-term industrial pollution on bacterial communities in urban river sediments, and thus contributes to the increasing knowledge of microbial ecology in the urban river sediments.

## Conclusion

This study revealed the composition and structure of bacterial communities and their co-occurrence patterns in different types of industrially polluted sediments. The composition of the dominant bacterial phyla was similar in each sampling location, but the relative abundance was different, and there were significant differences among different locations. Environmental factors, including metals (Cu, Zn and Cd) and nutritional factors (TN, TP and TOC), had significant effects on the composition of bacterial communities in different types of industrially polluted sediments (all *P* < 0.01). Although the relative abundance of highly connected taxa (such as the genera *Denitratisoma*, *Anaeromyxobacter* and *Candidatus_Microthrix*) in the co-occurrence networks was low (0.24% ~ 0.65%), they may play a pivotal role in maintaining the structure and function of ecological communities. Non-random co-occurrence and ecological function-driven modular patterns occurred in the bacterial communities, providing a new perspective of microbial assembly in different types of industrially polluted sediments. Furthermore, the accumulation of multiple chemical pollutant-degrading genes at the same location means that the zone was more polluted, while such functional response of bacterial community could contribute to the bioremediation of polluted river environment. Overall, these results provide valuable information for ecological risk assessment and management of urban rivers under different types of industrial pollution, thereby helping to monitor and control the level of water environmental contamination.

## Methods

### Sites description and sample collection

The sampling sites were located in the Qingliu River (watershed area of 1318 km^2^ and mainstream length of 84 km), which is a typical urban river in Chuzhou city, China [82]. The river flows through the center of the city and is surrounded by many industrial areas. Therefore, a large amount of different types of industrial wastewater is discharged along the river and is one of the main sources of pollution that causes the deterioration of the aquatic environment. Sampling points were from wastewater outlets of different types of factories near the Qingliu River (Fig. 1), which are respectively four different types of industrial pollution sources. The wastewater generated by these pollution sources is discharged into river water bodies through pipes. They are steel plant (GGS), lighting factory (ZMS), food factory (SPS) and textile mill (FZS), respectively. These factories are located on the outskirts of the city, and there are no other pollution sources in the surrounding areas.

Sampling was carried out in May 2019. A Peterson sampler was used to collect surface sediment samples (< 5 cm deep). Three parallel sediment samples were randomly obtained at 3 m intervals for each sampling site. The 4 sampling sites are shown in Fig. 1. The sediment samples were stored in sterile polyethylene zipper bags and transferred to the laboratory on ice within four hours. A portion of the sediment samples was collected into a 2.5 ml sterile centrifuge tubes and stored at − 80 °C until DNA extraction was performed. The other portion was immediately subjected to physio-chemical analysis at 4 °C.

### Analysis of physicochemical properties

While collecting the samples, the water temperature (T), pH and dissolved oxygen (DO) were measured in situ using a YSI-6600 multiparameter controller (Yellow Springs Instruments, USA). The ammonia nitrogen (NH_4_^+^-N), total nitrogen (TP), total phosphorus (TN), and total organic carbon (TOC) contents were measured in the laboratory according to standard methods [52]. The content of metal elements copper (Cu), zinc (Zn), lead (Pb), cadmium (Cd), chromium (Cr) in sediment samples were determined by using the X7 inductively coupled plasma mass spectrometer (ICP-MS) of Thermo Corporation in the collision cell mode [83].

### DNA extraction

Base on the manufacturer’s instructions, Total DNA was extracted from an aliquot of 0.25 g of sediment from each sample using an MP Biomedicals Fast DNATM SPIN Kit. The extracted genomic DNA was detected by 1% agarose gel electrophoresis, and the purity and concentration were determined by a UV spectrophotometer (Eppendorf, Germany). The measured DNA sample was stored at − 20 °C for subsequent use.

### PCR amplification and sequencing analysis

The V3-V4 region of the bacterial 16S rRNA gene was amplified using primers 338 F (5′-ACTCCTACGGGAGGCAGCAG-3′) and 806R (5′-GGACTACHVGGGTWTCTAAT-3′). All samples were analyzed in accordance with the formal experimental conditions with three replicates per sample. The PCR products were mixed and detected by 2% agarose gel electrophoresis. Then, based on the sequencing quantity requirements of each sample, the corresponding proportions were mixed. The samples were denatured with sodium hydroxide, and a single-stranded DNA fragment was finally obtained. The extracted DNA was then transported on ice to Shanghai Marobbio Biopharmaceutical Technology Co., Ltd. for sequencing.

Usearch (version 7.1 http://drive5.com/uparse/) was used to merged the paired-end 16S reads, trimmed primers and distal bases and removed quality-filtered sequences and singletons [84]. The RDP classifier (version 2.2, http://sourceforge.net/projects/rdp-classifier) Bayesian algorithm (confidence threshold of 0.7) was used to obtain the species classification information corresponding to each OTU, and OTU representative sequences were classified at a similarity level of 97% using the QIIME platform (http:// qiime.org/scripts/assign_taxonomy.html) [85].

### Statistical analysis

According to the OTU information, mothur (version v.1.30.1) was used to calculate the alpha diversity index (Chao 1, ACE, Shannon, Simpson) [86], and curves were generated with the R language tool [87]. Beta diversity analysis was represented by Bray-Curtis distance matrices generated from the OTU table, and statistical analysis and mapping was performed with PCoA in R language [88]. The community structure composition of different classification levels (such as phylum, genus and OTU) was obtained by taxonomic analysis. A similarity analysis (ANOSIM) of the Bray-Curtis similarity matrix of the initial pyrophosphate phosphate content was performed by the R-language vegan software package. One-way analysis of variance was performed using SPSS 19.0 and post-hoc Scheffe test was used for pairwise comparisons. The level of significance was set at 0.05. The relationship between microbial community composition and environmental factors was explained through the RDA function of the vegan package using R language [89]. The significantly discriminant taxa in each group were determined by the linear discriminant analysis (LDA) effect size pipeline (LEfSe) program at http://huttenhower.sph.harvard.edu/galaxy/root, which employs the factorial Kruskal-Wallis rank-sum test to identify communities or species that have significant differences in sample partitioning between species [90]. PICRUSt software was used to predict bacterial function and metabolic pathways [28], and the bioinformatics analysis images were drawn using the Origin software. Based on the Spearman correlation, the co-occurrence networks of microbial communities were determined. Select OTUs with significant and robust correlations (ρ > 0.7 and *P* < 0.05). All the robust correlations identified from pairwise comparison of the genera abundance form a correlation network where each node represents one genus, and each edge stands for a strong and significant correlation between the nodes. Gephi (http://gephi.github.io/) was used for network visualization and modularization analysis. The topological properties of the network, including degree (the number of neighbors of a node), average path length (the average number of steps along the shortest paths for all possible pairs of network nodes), clustering coefficient (he tendency of neighbors of a node to connect with each other), betweenness centrality (the number of shortest paths going through a node) and modularity (a measure of how well a network is divided into modules) were determined by gephi [91]. Nodes with high degree and low betweenness centrality values were recognized as keystone species in the co-occurrence network [35].

## Supplementary information

**Additional file 1: Table S1.** Physicochemical parameters of sediments and surface water for four sample sites. **Table S2.** Contamination levels of heavy metal concentrations in sediments at four sampling sites. **Table S3.** Estimates of richness and diversity for operational taxonomic units (OTUs) definition of 97% similarity for different types of industrial contaminated sediments from four sample sites. **Table S4.** ANOSIM test for differences among location groups. **Figure S1.** Pairwise comparison of the relative abundance of dominant bacteria in different industrial contaminated sediments. (a) Proteobacteria; (b) Actinobacteria; (c) Chloroflexi; (d) Acidobacteria; (e) Firmicutes; (f) Bacteroidetes. * *P* < 0.05, ** *P* < 0.01, *** *P* < 0.001. **Figure S2.** Indicator bacteria with LDA score of 3.5 or greater in bacterial communities associated with different industrial polluted sediments.

## Data Availability

The dataset generated and analysed in the current study is publicly available in NCBI’s Sequence Read Archive (SRA) repository under the BioProject ID PRJNA601066 (https://submit.ncbi.nlm.nih.gov/subs/sra/SUB6827030/overview).

## Abbreviations

LDA: Linear discriminant analysis
LEfSe: Linear discriminant analysis effect size
PCoA: Principal coordinate analysis
ANOSIM: Similarity analysis
RDA: Redundancy analysis
APL: Average path length
CC: Clustering coefficient
MD: Modularity index
OUT: Operational taxonomic units
PICRUSt: Phylogenetic investigation of communities by reconstruction of unobserved states
KEGG: Kyoto Encyclopedia of Genes and Genomes
SRB: Sulfate-reducing bacteria
T: Temperature
DO: Dissolved oxygen
TN: Total nitrogen
TP: Total phosphorus
TOC: Total organic carbon

## References

[1] Francis RA (2012). Positioning urban rivers within urban ecology. Urban Ecosyst.

[2] García-Armisen T, İnceoğlu Ö, Ouattara NK, Anzil A, Verbanck MA, Brion N, et al. Seasonal variations and resilience of bacterial communities in a sewage polluted urban river. PLoS One. 2014;9(3):e92579. 10.1371/journal.pone.0092579 PMC3965440 24667680

[3] Haller L, Tonolla M, Zopfi J, Peduzzi R, Wildi W, Pote J (2011). Composition of bacterial and archaeal communities in freshwater sediments with different contamination levels (Lake Geneva, Switzerland). Water Res.

[4] Echeveste P, Dachs J, Berrojalbiz N, Agustí S (2010). Decrease in the abundance and viability of oceanic phytoplankton due to trace levels of complex mixtures of organic pollutants. Chemosphere..

[5] Martínez-Santos M, Lanzén A, Unda-Calvo J, Martín I, Garbisu C, Ruiz-Romera E (2018). Treated and untreated wastewater effluents alter river sediment bacterial communities involved in nitrogen and Sulphur cycling. Sci Total Environ.

[6] Tani K, Masuhara M, Welikala N, Yamaguchi N, Nasu M (1998). Change in bacterial community during biodegradation of aniline. J Appl Microbiol.

[7] Sinclair RG. Wastewater irrigation and health: assessing and mitigating risk in low-income countries. Int J Water Resour D. 2010;26(4);704–9.

[8] Kuang J, Huang L, He Z, Chen L, Hua Z, Jia P (2016). Predicting taxonomic and functional structure of microbial communities in acid mine drainage. ISME J..

[9] Newton RJ, Jones SE, Eiler A, McMahon KD, Bertilsson S (2011). A guide to the natural history of freshwater lake bacteria. Microbiol Mol Biol Rev.

[10] Oikonomou A, Filker S, Breiner HW, Stoeck T (2015). Protistan diversity in a permanently stratified meromictic Lake (Lake a latsee, SW G ermany). Environ Microbiol.

[11] Guo X-p, Lu D-p, Z-s N, J-n F, Y-r C, F-y T (2018). Bacterial community structure in response to environmental impacts in the intertidal sediments along the Yangtze estuary. China Mar Pollut Bull.

[12] Zhang W, Li Y, Wang C, Wang P, Hou J, Yu Z (2016). Modeling the biodegradation of bacterial community assembly linked antibiotics in river sediment using a deterministic–stochastic combined model. Environ Sci Technol.

[13] Wang J, Li Y, Wang P, Niu L, Zhang W, Wang C (2016). Response of bacterial community compositions to different sources of pollutants in sediments of a tributary of Taihu Lake. China Environ Sci Pollut Res.

[14] Guo X-p, Yang Y, Niu Z-S, Lu D-P, Zhu C-H, Feng J-N (2019). Characteristics of microbial community indicate anthropogenic impact on the sediments along the Yangtze estuary and its coastal area, China. Sci Total Environ.

[15] Ducrotoy J-P (2010). The use of biotopes in assessing the environmental quality of tidal estuaries in Europe. Estuar Coast Shelf Sci.

[16] Li D, Sharp JO, Drewes JE (2016). Influence of wastewater discharge on the metabolic potential of the microbial community in river sediments. Microb Ecol.

[17] Rasool A, Xiao T (2018). Response of microbial communities to elevated thallium contamination in river sediments. Geomicrobiol J.

[18] Liao K, Bai Y, Huo Y, Jian Z, Hu W, Zhao C (2018). Integrating microbial biomass, composition and function to discern the level of anthropogenic activity in a river ecosystem. Environ Int.

[19] Hu A, Ju F, Hou L, Li J, Yang X, Wang H (2017). Strong impact of anthropogenic contamination on the co-occurrence patterns of a riverine microbial community. Environ Microbiol.

[20] Ma B, Wang H, Dsouza M, Lou J, He Y, Dai Z (2016). Geographic patterns of co-occurrence network topological features for soil microbiota at continental scale in eastern China. ISME J..

[21] Barberán A, Bates ST, Casamayor EO, Fierer N (2012). Using network analysis to explore co-occurrence patterns in soil microbial communities. ISME J.

[22] Faust K, Raes J (2012). Microbial interactions: from networks to models. Nat Rev Microbiol.

[23] Li Y, Wu H, Shen Y, Wang C, Wang P, Zhang W (2019). Statistical determination of crucial taxa indicative of pollution gradients in sediments of Lake Taihu. China Environ Pollut.

[24] Gilbert JA, Steele JA, Caporaso JG, Steinbrück L, Reeder J, Temperton B (2012). Defining seasonal marine microbial community dynamics. ISME J..

[25] Xue L, Ren H, Li S, Leng X, Yao X (2017). Soil bacterial community structure and co-occurrence pattern during vegetation restoration in karst rocky desertification area. Front Microbiol.

[26] Lu L, Yin S, Liu X, Zhang W, Gu T, Shen Q (2013). Fungal networks in yield-invigorating and-debilitating soils induced by prolonged potato monoculture. Soil Biol Biochem.

[27] He D, Shen W, Eberwein J, Zhao Q, Ren L, Wu QL (2017). Diversity and co-occurrence network of soil fungi are more responsive than those of bacteria to shifts in precipitation seasonality in a subtropical forest. Soil Biol Biochem.

[28] Langille MG, Zaneveld J, Caporaso JG, McDonald D, Knights D, Reyes JA (2013). Predictive functional profiling of microbial communities using 16S rRNA marker gene sequences. Nat Biotechnol.

[29] Ceballos B, Soares N, Moraes M, Catão R, Konig A (2003). Microbiological aspects of an urban river used for unrestricted irrigation in the semi-arid region of north-East Brazil. Water Sci Technol.

[30] Ouattara NK, Passerat J, Servais P (2011). Faecal contamination of water and sediment in the rivers of the Scheldt drainage network. Environ Monit Assess.

[31] Servais P, Garcia-Armisen T, George I, Billen G (2007). Fecal bacteria in the rivers of the seine drainage network (France): sources, fate and modelling. Sci Total Environ.

[32] Yang Z, Yu T, Hou Q, Xia X, Feng H, Huang C (2014). Geochemical evaluation of land quality in China and its applications. J Geochem Explor.

[33] Newman ME (2003). The structure and function of complex networks. SIAM Rev.

[34] Newman ME (2006). Modularity and community structure in networks. Proc Natl Acad Sci.

[35] Berry D, Widder S (2014). Deciphering microbial interactions and detecting keystone species with co-occurrence networks. Front Microbiol.

[36] Ren Z, Wang F, Qu X, Elser JJ, Liu Y, Chu L (2017). Taxonomic and functional differences between microbial communities in Qinghai lake and its input streams. Front Microbiol.

[37] Araya R, Tani K, Takagi T, Yamaguchi N, Nasu M (2003). Bacterial activity and community composition in stream water and biofilm from an urban river determined by fluorescent in situ hybridization and DGGE analysis. FEMS Microbiol Ecol.

[38] Wang P, Chen B, Yuan R, Li C, Li Y (2016). Characteristics of aquatic bacterial community and the influencing factors in an urban river. Sci Total Environ.

[39] Su Z, Dai T, Tang Y, Tao Y, Huang B, Mu Q (2018). Sediment bacterial community structures and their predicted functions implied the impacts from natural processes and anthropogenic activities in coastal area. Mar Pollut Bull.

[40] Carozza L, Berger JF, Burenscarozza A, Cyril M. Microbial Surface Colonization and Biofilm Development in Marine Environments. Microbiol Mol Biol R Mmbr. 2015;80(1):91. 10.1128/MMBR.00037-15 PMC4711185 26700108

[41] Smalley NE, Taipale S, De Marco P, Doronina NV, Kyrpides N, Shapiro N (2015). Functional and genomic diversity of methylotrophic Rhodocyclaceae: description of Methyloversatilis discipulorum sp. nov. Int J Syst Evol Microbiol.

[42] Oren A. The Family Rhodocyclaceae. In: The Prokaryotes. Heidelberg: Springer; 2014. p. 975–98.

[43] Köchling T, Sanz JL, Galdino L, Florencio L, Kato MT (2017). Impact of pollution on the microbial diversity of a tropical river in an urbanized region of northeastern Brazil. Int Microbiol.

[44] Mikhailov IS, Zakharova YR, Bukin YS, Galachyants YP, Petrova DP, Sakirko MV (2019). Co-occurrence networks among bacteria and microbial eukaryotes of Lake Baikal during a spring phytoplankton bloom. Microb Ecol.

[45] Wang L, Zhang J, Li H, Yang H, Peng C, Peng Z (2018). Shift in the microbial community composition of surface water and sediment along an urban river. Sci Total Environ.

[46] Basta T, Buerger S, Stolz A (2005). Structural and replicative diversity of large plasmids from sphingomonads that degrade polycyclic aromatic compounds and xenobiotics. Microbiology..

[47] Glaeser SP, Grossart HP, Glaeser J (2010). Singlet oxygen, a neglected but important environmental factor: short-term and long-term effects on bacterioplankton composition in a humic lake. Environ Microbiol.

[48] Sprenger GA (1993). Approaches to broaden the substrate and product range of the ethanologenic bacterium Zymomonas mobilis by genetic engineering. J Biotechnol.

[49] Jordaan K, Bezuidenhout C (2016). Bacterial community composition of an urban river in the north West Province, South Africa, in relation to physico-chemical water quality. Environ Sci Pollut Res.

[50] Ghosh M, Verma S, Mengoni A, Tripathi A (2004). Enrichment and identification of bacteria capable of reducing chemical oxygen demand of anaerobically treated molasses spent wash. J Appl Microbiol.

[51] Belli T, Battistelli A, Costa R, Vidal C, Schlegel A, Lapolli F (2019). Evaluating the performance and membrane fouling of an electro-membrane bioreactor treating textile industrial wastewater. Int J Environ Sci Te.

[52] Wu H, Li Y, Zhang J, Niu L, Zhang W, Cai W (2017). Sediment bacterial communities in a eutrophic lake influenced by multiple inflow-rivers. Environ Sci Pollut Res.

[53] Rebollar EA, Sandoval-Castellanos E, Roessler K, Gaut BS, Alcaraz LD, Benítez M (2017). Seasonal changes in a maize-based polyculture of Central Mexico reshape the co-occurrence networks of soil bacterial communities. Front Microbiol.

[54] Jacquiod S, Cyriaque V, Riber L, Al-Soud WA, Gillan DC, Wattiez R (2018). Long-term industrial metal contamination unexpectedly shaped diversity and activity response of sediment microbiome. J Hazard Mater.

[55] Biddanda B, Ogdahl M, Cotner J (2001). Dominance of bacterial metabolism in oligotrophic relative to eutrophic waters. Limnol Oceanogr.

[56] Ibekwe AM, Ma J, Murinda SE (2016). Bacterial community composition and structure in an Urban River impacted by different pollutant sources. Sci Total Environ.

[57] Yannarell AC, Triplett EW (2005). Geographic and environmental sources of variation in lake bacterial community composition. Appl Environ Microbiol.

[58] Wang L, Liu X, Yu S, Shi X, Wang X, Zhang X-H (2017). Bacterial community structure in intertidal sediments of Fildes peninsula, maritime Antarctica. Polar Biol.

[59] Jansson M, Bergström A-K, Lymer D, Vrede K, Karlsson J (2006). Bacterioplankton growth and nutrient use efficiencies under variable organic carbon and inorganic phosphorus ratios. Microb Ecol.

[60] Wang NF, Zhang T, Yang X, Wang S, Yu Y, Dong LL (2016). Diversity and composition of bacterial community in soils and lake sediments from an arctic lake area. Front Microbiol.

[61] Chen Y, Jiang Y, Huang H, Mou L, Ru J, Zhao J (2018). Long-term and high-concentration heavy-metal contamination strongly influences the microbiome and functional genes in Yellow River sediments. Sci Total Environ.

[62] Chaffron S, Rehrauer H, Pernthaler J, Von Mering C (2010). A global network of coexisting microbes from environmental and whole-genome sequence data. Genome Res.

[63] Hu Y, Bai C, Cai J, Dai J, Shao K, Tang X, et al. Co-occurrence network reveals the higher fragmentation of the bacterial community in Kaidu River than its tributaries in Northwestern China. Microbes Environ. 2018;33(2):127–34. 10.1264/jsme2.ME17170 PMC6031398 29794413

[64] Bian W, Li J, Hou A, Wang M, Zhang S (2016). Rapidly startup of partial nitrification in sequencing batch reactor and microbiological analysis. Desalin Water Treat.

[65] Marshall MJ, Dohnalkova AC, Kennedy DW, Plymale AE, Thomas SH, Löffler FE (2009). Electron donor-dependent radionuclide reduction and nanoparticle formation by Anaeromyxobacter dehalogenans strain 2CP-C. Environ Microbiol.

[66] Wu Q, Sanford RA, Löffler FE (2006). Uranium (VI) reduction by Anaeromyxobacter dehalogenans strain 2CP-C. Appl Environ Microbiol.

[67] Maza-Márquez P, Castellano-Hinojosa A, González-Martínez A, Juárez-Jiménez B, González-López J, Rodelas B (2019). Abundance of total and metabolically active Candidatus Microthrix and fungal populations in three full-scale wastewater treatment plants. Chemosphere..

[68] Olesen JM, Bascompte J, Dupont YL, Jordano P (2007). The modularity of pollination networks. Proc Natl Acad of Sci.

[69] Gesheva V, Vasileva-Tonkova E (2012). Production of enzymes and antimicrobial compounds by halophilic Antarctic Nocardioides sp. grown on different carbon sources. World J Microbiol Biotechnol.

[70] Song W, Qi R, Zhao L, Xue N, Wang L, Yang Y (2019). Bacterial community rather than metals shaping metal resistance genes in water, sediment and biofilm in lakes from arid northwestern China. Environ Pollut.

[71] Jiao S, Liu Z, Lin Y, Yang J, Chen W, Wei G (2016). Bacterial communities in oil contaminated soils: biogeography and co-occurrence patterns. Soil Biol Biochem.

[72] Zhang Y. Isolation of sulfate-reducing Bacteria from heavy metal-contaminated sediments and their ability to reduce arsenate abstract: Shenyang Normal University. 2015. https://kns.cnki.net/KCMS/detail/detail.aspx?dbcode=CMFD&dbname=CMFD201502&filename=1015309291.nh&uid=WEEvREcwSlJHSldRa1FhcTdnTnhVaGQxQndhZUZvNC8yWno5UUFJZ1libz0=$9A4hF_YAuvQ5obgVAqNKPCYcEjKensW4IQMovwHtwkF4VYPoHbKxJw!!&v=MjEzMTViUElSOGVYMUx1eFlTN0RoMVQzcVRyV00xRnJDVVI3cWZiK1puRkNqZ1Y3M09WRjI2RzdDNEY5UEZycEU=. .

[73] Cao J, Li Y, Zhang G, Yang C, Cao X (2013). Effect of Fe (III) on the biotreatment of bioleaching solutions using sulfate-reducing bacteria. Int J Miner Process.

[74] Zhou Q, Chen Y, Yang M, Li W, Deng L (2013). Enhanced bioremediation of heavy metal from effluent by sulfate-reducing bacteria with copper–iron bimetallic particles support. Bioresour Technol.

[75] Kansole MM, Lin T-F (2016). Microcystin-LR biodegradation by Bacillus sp.: reaction rates and possible genes involved in the degradation. Water.

[76] Yergeau E, Sanschagrin S, Beaumier D, Greer CW. Metagenomic analysis of the bioremediation of diesel-contaminated Canadian high arctic soils. PLoS One. 2012;7(1):e30058. 10.1371/journal.pone.0030058 PMC3256217 22253877

[77] Bai Y, Qi W, Liang J, Qu J (2014). Using high-throughput sequencing to assess the impacts of treated and untreated wastewater discharge on prokaryotic communities in an urban river. Appl Microbiol Biotechnol.

[78] Gan HM, Hudson AO, Rahman AYA, Chan KG, Savka MA (2013). Comparative genomic analysis of six bacteria belonging to the genus Novosphingobium: insights into marine adaptation, cell-cell signaling and bioremediation. BMC Genomics.

[79] Wang Y, Wu Y, Wu Z, Tam NF-Y (2015). Genotypic responses of bacterial community structure to a mixture of wastewater-borne PAHs and PBDEs in constructed mangrove microcosms. J Hazard Mater.

[80] Xu X, Chen X, Su P, Fang F, Hu B (2016). Biodegradation potential of polycyclic aromatic hydrocarbons by bacteria strains enriched from Yangtze River sediments. Environ Technol.

[81] Roberto AA, Van Gray JB, Leff LG (2018). Sediment bacteria in an urban stream: spatiotemporal patterns in community composition. Water Res.

[82] Zhang H. Analysis of runoff evolution based on data mining under changing environment: University of Electronic Science and Technology of China. 2018. https://kns.cnki.net/KCMS/detail/detail.aspx?dbcode=CMFD&dbname=CMFD201802&filename=1018991416.nh&uid=WEEvREcwSlJHSldRa1FhcTdnTnhVaGQxQndhZUZvNC8yWno5UUFJZ1libz0=$9A4hF_YAuvQ5obgVAqNKPCYcEjKensW4IQMovwHtwkF4VYPoHbKxJw!!&v=MTMwNTZyV00xRnJDVVI3cWZiK1puRkNqaFc3ck1WRjI2RnJxeEg5WE5xWkViUElSOGVYMUx1eFlTN0RoMVQzcVQ=.

[83] Wu E, Zhao J, Qiao X, Dong Y, Gao Y (2014). Characteristics and pollution assessment of surface sediments of heavy metals in Hutuo River. J Shihezi Univ (Nat Sci).

[84] Edgar RC (2010). Search and clustering orders of magnitude faster than BLAST. Bioinformatics..

[85] Caporaso JG, Kuczynski J, Stombaugh J, Bittinger K, Bushman FD, Costello EK (2010). QIIME allows analysis of high-throughput community sequencing data. Nat Methods.

[86] Shao J, He Y, Zhang H, Chen A, Lei M, Chen J (2016). Silica fertilization and nano-MnO 2 amendment on bacterial community composition in high arsenic paddy soils. Appl Microbiol Biotechnol.

[87] Team RC (2013). R: a language and environment for statistical computing.

[88] Mitter EK, de Freitas JR, Germida JJ (2017). Bacterial root microbiome of plants growing in oil sands reclamation covers. Front Microbiol.

[89] Oksanen J, Blanchet G, Friendly M, Kindt R, Legendre P, McGlinn D, et al. vegan: Community Ecology Package: R package version 2.5–2. 2018. https://cran.r-project.org/package=vegan.

[90] Segata N, Izard J, Waldron L, Gevers D, Miropolsky L, Garrett WS (2011). Metagenomic biomarker discovery and explanation. Genome Biol.

[91] Bastian M, Heymann S, Jacomy M (2009). Gephi: an open source software for exploring and manipulating networks. Third international AAAI conference on weblogs and social media.

