# Supplementary material for: Impact of long-term industrial contamination on the bacterial communities in urban river sediments
Source: BMC Microbiol. 2020 Aug 14;20:254. doi: 10.1186/s12866-020-01937-x (PMC7427966; doi:10.1186/s12866-020-01937-x)
Supplement: Supplementary file 1 — Additional file 1: Table S1. Physicochemical parameters of sediments and surface water for four sample sites. Table S2. Contamination levels of heavy metal concentrations in sediments at four sampling sites. Table S3. Estimates of richness and diversity for operational taxonomic units (OTUs) definition of 97% similarity for different types of industrial contaminated sediments from four sample sites. Table S4. ANOSIM test for differences among location groups. Figure S1. Pairwise comparison of the relative abundance of dominant bacteria in different industrial contaminated sediments. (a) Proteobacteria; (b) Actinobacteria; (c) Chloroflexi; (d) Acidobacteria; (e) Firmicutes; (f) Bacteroidetes. * P < 0.05, ** P < 0.01, *** P < 0.001. Figure S2. Indicator bacteria with LDA score of 3.5 or greater in bacterial communities associated with different industrial polluted sediments. [file 12866_2020_1937_MOESM1_ESM.docx]

**Supplementary materials**

**Table S1.** Physicochemical parameters of sediments and surface water for four sample sites.

| **Physicochemical parameters** | | | | | | | | | | | | | | |  |  |
| --- | --- | --- | --- | --- | --- | --- | --- | --- | --- | --- | --- | --- | --- | --- | --- | --- |
| **Surface water** | | | | |  | **Sediment** | | | | | | | | | |  |
| **Samples** | **T**  **(°C)** | **pH** | **Do**  **(mg/L)** | **NH_4_^+^-N**  **(mg/L)** |  | **Samples** | **TN**  **(mg/g, dry mass)^a^** | **TP**  **(mg/g, dry mass)^a^** | **TOC**  **(mg/g, dry mass)^a^** | **Cu**  **(mg/kg, dry mass)^a^** | **Zn**  **(mg/kg, dry mass)^a^** | **Pb**  **(mg/kg, dry mass)^a^** | **Cd**  **(mg/kg, dry mass)^a^** | **Cr**  **(mg/kg, dry mass)^a^** | | |
| GGW | 22.7 | 7.49 | 8.72±0.03 | 1.00±0.08 |  | GGS | 4.88±0.02 | 2.83±0.02 | 10.67±0.08 | 123.03 ±5.80 | 382.92 ±12.18 | 60.12 ±5.61 | 0.28 ±0.02 | 74.65 ±5.94 | | |
| ZMW | 21.2 | 7.53 | 8.77±0.06 | 1.02±0.10 |  | ZMS | 2.48±0.10 | 1.39±0.01 | 4.27±0.14 | 82.12 ± 3.89 | 256.38 ± 10.24 | 178.01 ± 6.28 | 1.83 ± 0.16 | 87.35 ± 5.29 | | |
| SPW | 21.3 | 7.24 | 8.92±0.03 | 1.11±0.13 |  | SPS | 2.24±0.05 | 1.76±0.07 | 3.17±0.03 | 52.88 ± 2.74 | 122.59 ± 8.15 | 71.79 ± 2.55 | 1.57 ± 0.12 | 56.95 ± 3.50 | | |
| FZW | 22.1 | 9.96 | 8.83±0.12 | 0.82±0.05 |  | FZS | 1.65±0.06 | 0.63±0.02 | 2.78±0.11 | 94.09 ± 6.34 | 262.52 ± 6.19 | 62.50 ± 3.34 | 3.60 ± 0.39 | 68.53 ± 4.38 | | |

Note: T, temperature; DO, dissolved oxygen; NH_4_^+^-N, ammonia nitrogen; TN, total nitrogen; TP, total phosphorus; TOC, total organic carbon; Zn, zinc; Cu, copper; Pb, lead; Cd, cadmium; Cr, chromium. a Values are given as mean ± standard deviation.

**Table S2.** Contamination levels of heavy metal concentrations in sediments at four sampling sites

| **Samples** | **Heavy metal** | | | | |
| --- | --- | --- | --- | --- | --- |
|  | Cu | Zn | Pb | Cd | Cr |
| GGS | Ⅲ | Ⅲ | Ⅱ | Ⅱ | Ⅰ |
| ZMS | Ⅱ | Ⅲ | Ⅱ | Ⅳ | Ⅰ |
| SPS | Ⅱ | Ⅱ | Ⅱ | IV | Ⅰ |
| FZS | Ⅱ | Ⅱ | Ⅱ | Ⅴ | Ⅰ |

Note: According to the Environmental Quality standards for Soils of China (GB15618- 1995), concentrations of heavy metals are classified into five classes (I, II, III, IV, and V, which correspond to clean, relatively clean, normal, polluted, and moderately to heavily polluted, respectively).

**Table S3.** Estimates of richness and diversity for operational taxonomic units (OTUs) definition of 97% similarity for different types of industrial contaminated sediments from four sample sites.

| **Samples** | **Sequences** | **OTUs** | **Chao 1** | **ACE** | **Simpson** | **Shannon** | **coverage** |
| --- | --- | --- | --- | --- | --- | --- | --- |
| GGS1 | 31412 | 737 | 844.78 | 830.18 | 0.0174 | 5.09 | 0.994 |
| GGS2 | 42236 | 715 | 864.86 | 812.78 | 0.0218 | 4.90 | 0.994 |
| GGS3 | 42481 | 708 | 813.92 | 791.45 | 0.0194 | 4.94 | 0.995 |
| SPS1 | 35160 | 1116 | 1298.89 | 1246.89 | 0.0152 | 5.59 | 0.991 |
| SPS2 | 40386 | 1141 | 1280.94 | 1249.26 | 0.0170 | 5.59 | 0.992 |
| SPS3 | 33796 | 1115 | 1287.33 | 1230.45 | 0.0200 | 5.56 | 0.992 |
| FZS1 | 53831 | 950 | 1027.00 | 995.40 | 0.0058 | 5.96 | 0.996 |
| FZS2 | 54342 | 893 | 974.58 | 934.80 | 0.0045 | 6.01 | 0.996 |
| FZS3 | 48180 | 915 | 1024.00 | 971.60 | 0.0053 | 5.97 | 0.995 |
| ZMS1 | 44184 | 1145 | 1286.92 | 1252.86 | 0.0069 | 5.92 | 0.992 |
| ZMS2 | 38863 | 1150 | 1231.88 | 1209.78 | 0.0078 | 5.96 | 0.994 |
| ZMS3 | 41040 | 1135 | 1277.92 | 1223.57 | 0.0066 | 6.01 | 0.993 |

**Table S4.** ANOSIM test for differences among location groups.

| **Groups** | **r (Correlation)** | **P (Significance)** | **Permutation Number** |
| --- | --- | --- | --- |
| All | 0.8951 | 0.001 | 999 |

**Fig. S1.** Pairwise comparison of the relative abundance of dominant bacteria in different industrial contaminated sediments. (a) Proteobacteria; (b) Actinobacteria; (c) Chloroflexi; (d) Acidobacteria; (e) Firmicutes; (f) Bacteroidetes. * P <0.05, ** P <0.01, *** P <0.001.





Fig. S2. Indicator bacteria with LDA score of 3.5 or greater in bacterial communities associated with different industrial polluted sediments.
